# Supplementary material for: From labels to priors in capsule endoscopy: a prior guided approach for improving generalization with few labels
Source: Sci Rep. 2022 Sep 20;12:15708. doi: 10.1038/s41598-022-19675-7 (PMC9489743; doi:10.1038/s41598-022-19675-7)
Supplement: Supplementary file 1 — Supplementary Information. [file 41598_2022_19675_MOESM1_ESM.pdf]

1 Additional Preprocessing and Training Details

1.1 Normalization

Gastrointestinal color space can be very different from natural images, therefore we compute the mean and standard deviation for this study on our train set (PS-DeVCEM, OSF-Kvasir datasets). The RGB mean and standard deviation used in this work are [0.56, 0.35, 0.20] and [0.3, 0.24, 0.17] respectively.

1.2 Transformation $\mathscr{T}_p$, $\mathscr{T}_d$ and $\mathscr{T}_{win}$

Transformation are applied using PyTorch and Python Imaging Library. $\mathscr{T}_p$ applied to v_p^i and the mutual tiles from v_d^i is color-jittering (brightness=0.4, contrast=0.4, saturation=0.4, hue=0) and random horizontal and vertical flipping. For PIRL we use the augmentation in [1] except that we consider a fixed crop (eliminating borders) instead of random crop to have a more controlled setting for studying the benefit from views. (Th effect of random crops is studied separately as Rand PGCon in Table 4.) $\mathscr{T}_d$ is applied to v_d^i and is different from [1] as it distorts the tiles selectively. $\mathscr{T}_d$ includes erasing with probability 0.5, gray-scaling, color-jittering (brightness=0.4, contrast=0.4, saturation=0.4, hue=0) as well as vertical and horizontal flips. For the within instance negatives, a stronger augmentation was applied as it was found to be a hard negative for the corresponding v_p^i. We used RandAugment policy (`https://github.com/kakaobrain/fast-autoaugment`) with $n = 1$ i.e., one distortion per image and m is random on scale (0,30). The applied distortions are one of brightness, contrast, sharpness, equalize, rotate, posterize, solarize, shearx, sheary. Since this is a negative already, we do not use random erasing here. We train on two separate resources subject to availability, a Twin-Titan RTX (2 GPUs, 24GB each) and a shared GPU-cluster [2].

2 Evaluation and Ablation

2.1 Linear Evaluation:

We perform linear evaluation using SGD optimizer with a initial and final learning rate [0.1,12e-5] respectively with cosine annealing, momentum 0.9, weight decay of $1e-4$ and batch size 64. We train for 300 epochs for 100% data, 200 epoch for 10% and approx 100 epochs for 1% data. The data augmentation for linear evaluation includes centre crop, PyTorch color jitter with (brightness=0.4, contrast=0.6, saturation=0, hue=0), random horizontal and vertical flipping. We train the BatchNorm layers during linear evaluation as it provides slightly better or equal performance.

2.2 Fine Tuning

For finetuning we follow the same setup, augmentation and hyperparameters as linear evaluation.

2.3 Ablation: Quantifying the gain from domain priors

We evaluate the gain accrued from using the two priors (redness and locality) over PIRL baseline. PIRL uses neither locality nor preference for specific regions within images. Rand PGCon is a variant that uses a random v_p from within the image such that it is a subregion but not strategically chosen, therefore it promotes only locality, not redness. PGCon uses both locality as well as redness priors. Table 1 shows representations in WCE benefit from both priors.

**Gain from enforcing Prior guided attention**	Locality	Redness	1%	10%	100%
PIRL	-	-	29.6	50.7	88.7
Rand PGCon	✓	-	43.9 (+14)	59.3 (+8)	89.0 (+0.3)
Locality + Redness (PGCon)	✓	✓	52.9 (+9)	75.1 (+15)	91.0 (+2)

Table 1. Ablation: Gain from priors. Table shows result of linear evaluation on CAD-CAP.

2.4 Ablation: Projection head

As [3], we replace the linear projection head with an MLP head (additional hidden layer 1024d, ReLU activation) only in contrastive pretraining. We do not observe any significant increase in performance from transferring the representation from MLP head compared to linear head for downstream classification on CAD-CAP.

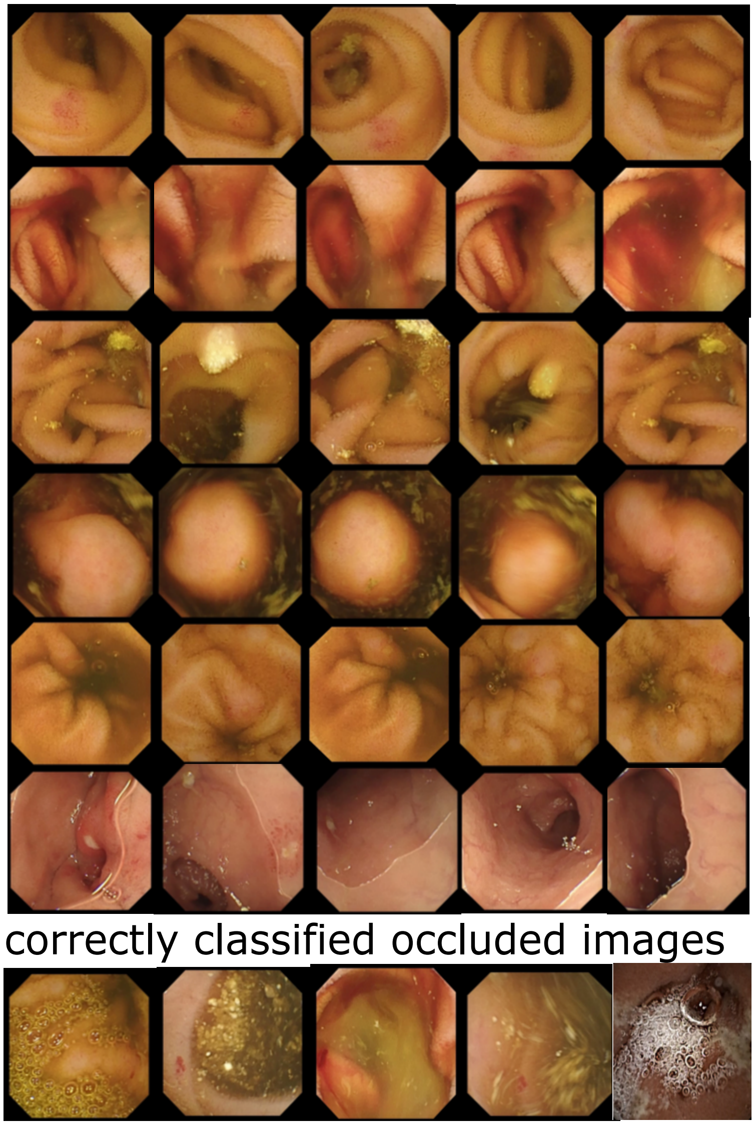

Figure 1. Figure (all but last row) illustrates the nearest neighbors in space based on euclidean distance. The last row shows robustness to domain artifacts, examples of images correctly classified despite occlusions in images.

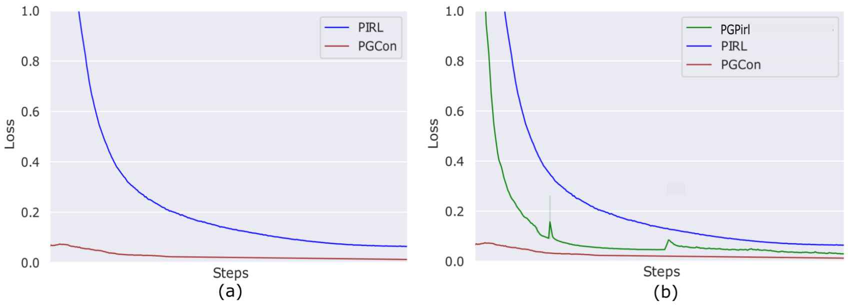

Figure 2. The effect of prior guided views leads to a faster convergence than PIRL, with further switching v_p as the anchor lowers the initial loss substantially.

Method	Epochs	Batch size	Aug	CAD-CAP Top-1 Accuracy
P-Con	600	64	T_p, T_d	**94.7**
PG-Con MLP	600	64	T_p, T_d	93

Table 2. Projection head ablation: Both networks use R50 backbone.

3 Uniformity and Alignment Analysis

We use the implementation of alignment $\mathscr{L}_{align}$ and uniformity loss $\mathscr{L}_{uniform}$ in [4] to evaluate our encoders. The features z_p and z_d are L2-normalized and $\alpha = t = 2$. $\mathscr{L}_{align}$ is computed between pairs z_p, z_d for all models. Uniformity for PGCon, PIRL and ImageNet-pretrained encoder is computed over embeddings z_p and z_d therefore averaging over $\mathscr{L}_{uniform}(z_p)$ and $\mathscr{L}_{uniform}(z_d)$ in a batch. In WINCon however, the WINs need to be considered additionally to z_p and z_d for computing overall uniformity. Therefore, $\mathscr{L}_{uniform-win}$ is averaged over $\mathscr{L}_{uniform}(z_p)$, $\mathscr{L}_{uniform}(z_d)$ and $\mathscr{L}_{uniform}(z_{win})$.

4 Improvement over PIRL [1]

The main objective in PIRL is to learn representations despite transformations instead of through them. This idea is powerful and has inspired our work, however in this work we use the PIRL setup as a tool to guide additional invariances towards specialized representations. The first difference is in the objective (written in PIRL style, refer (4), (2) (3)). In PIRL, the anchor is R_i which is an evolving average for original instance i stored in $\mathscr{M}$ and is contrasted with transformed and original images, there isn't any explicit view priority. For us however, the anchor is a pathology region v_p^i. The difference in the two anchors is that, in our case, the memory in each iteration will be updated with (v_p) which is a twice contrasted (against 2k negatives) view (3). The result of this seemingly simple switching can be seen on the contrastive loss in Fig. 2 (a) and (b). $\mathscr{L}_{Pirl}$ corresponds to the PIRL objective, $\mathscr{L}_{PGPirl}$ refers to the variant when we use our prior guided views v_p and v_d in PIRL objective without anchor switching. Lastly, $\mathscr{L}_{PGCon}$ shows our objective with both anchor-switching as well as prior guided views.

$$L_{Pirl} = \alpha \mathscr{L}_{nce}(R_i, f(v_i^t)) + \beta \mathscr{L}_{nce}(R_i, h(v_i)) \tag{1}$$

$$L_{PGPirl} = \alpha \mathscr{L}_{nce}(R_i, f(v_i^d)) + \beta \mathscr{L}_{nce}(R_i, h(v_i^p)) \tag{2}$$

$$L_{PGCon} = \alpha \mathscr{L}_{nce}(f(v_i^p), R_i)) + \beta \mathscr{L}_{nce}(f(v_i^p), h(v_i^d)) \tag{3}$$

Supplementary Video

The attached supplementary video shows weak labels from our approach on a variety of images with different pathologies, both in small and large bowel, at varying bowel cleanliness levels.

Data Availability

The datasets OSF Kvasir is available at url: https://osf.io/dv2ag/#:~:text=This%20is%20the%20official%20OSF,and%20pathological%20and%20normal%20findings
KID, KID2 are publicly available at url : https://mdss.uth.gr/datasets/endoscopy/kid/

References

1. Misra, I. & Maaten, L. v. d. Self-supervised learning of pretext-invariant representations. In *Proceedings of the IEEE/CVF Conference on Computer Vision and Pattern Recognition*, 6707–6717 (2020).

2. Själander, M., Jahre, M., Tufte, G. & Reissmann, N. EPIC: An energy-efficient, high-performance GPGPU computing research infrastructure (2019). 1912.05848.

3. Chen, T., Kornblith, S., Norouzi, M. & Hinton, G. A simple framework for contrastive learning of visual representations. In *International conference on machine learning*, 1597–1607 (PMLR, 2020).

4. Wang, T. & Isola, P. Understanding contrastive representation learning through alignment and uniformity on the hypersphere (2020). 2005.10242.

Pytorch-style data augmentation for PGCon

```
# Prior- view Augmentation
torchvision.transforms.Resize((120, 120))(x)

ColorJitter(brightness=0.4, contrast=0.4, saturation=0.4, hue=0)(x)
RandomHorizontalFlip(p=0.5)(x)
RandomVerticalFlip(p=0.5)(x)
```

T_p

```
# Distorted - view Augmentation
# Random crop each tile to break edge continuity
# discontinuity ranges from 3 to 40 pixels between tiles
[RandomCrop((90, 90))(x) for x in jigsaw_tiles]
[ColorJitter(brightness=0.4, contrast=0.4, saturation=0.4, hue=0)(x) for x in jigsaw_tiles]
[RandomHorizontalFlip(p=0.5)(x) for x in jigsaw_tiles]
[RandomVerticalFlip(p=0.5)(x) for x in jigsaw_tiles]
# Random grayscale and erase from tiles not-mutual with prior-view
[RandomGrayscale(p=0.1) ))(x) for x in not_mutual]
[RandomErasing(p=0.5,scale=(0.02, 0.3), ratio=(0.3,2))(x) for x in not_mutual]
random.shuffle()
```

T_d

Figure 3. PGCon data augmentation

Pytorch-style dataaugmentation for WINCon

```
# Prior- view Augmentation
torchvision.transforms.Resize((120, 120))(x)
ColorJitter(brightness=0.4, contrast=0.4, saturation=0.4, hue=0)(x)     } T_p
RandomHorizontalFlip(p=0.5)(x)
RandomVerticalFlip(p=0.5)(x)
# Distorted - view Augmentation
# Random crop each tile to break edge continuity
# discontinuity ranges from 3 to 40 pixels between tiles
[RandomCrop((90, 90))(x) for x in jigsaw_tiles]
[ColorJitter(brightness=0.4, contrast=0.4, saturation=0.4, hue=0)(x) for x in jigsaw_tiles]   } T_d
[RandomHorizontalFlip(p=0.5)(x) for x in jigsaw_tiles]
[RandomVerticalFlip(p=0.5)(x) for x in jigsaw_tiles]

# Random grayscale and erase from tiles not-mutual with prior-view
[RandomGrayscale(p=0.1) ))(x) for x in not_mutual]
[RandomErasing(p=0.5,scale=(0.02, 0.3), ratio=(0.3,2))(x) for x in not_mutual]
random.shuffle()
# Within Instance Augmentation
torchvision.transforms.Resize((224, 224))(x)     } T_win
RandAugment(n=1,m=random.randint(0,30,1))
```

Figure 4. WINCon data augmentation
